# Supplementary material for: Metamitron, a Photosynthetic Electron Transport Chain Inhibitor, Modulates the Photoprotective Mechanism of Apple Trees
Source: Plants (Basel). 2021 Dec 17;10(12):2803. doi: 10.3390/plants10122803 (PMC8707989; doi:10.3390/plants10122803)
Supplement: Supplementary file 1 [file plants-10-02803-s001.zip › Supplementary Figure 1.pdf]

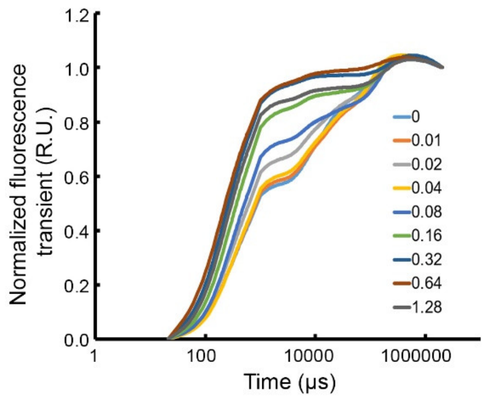


**Figure S1.** Brevis® active ingredient Metamitron obtains a PSII acceptor side inhibition profile in OJIP measurement in "Top-Red" cultivar. Upon gradient increase in Brevis's concentration, a saturation profile in the first electronic transition can be seen. Each curve is an average of three biological repeats. The concentrations gradient are presented in percentage. Fluorescence transient profiles were normalized to the maximum signal per each sample.
